# The epidemiology of cryptorchidism and potential risk factors, including endocrine disrupting chemicals

**DOI:** 10.3389/fendo.2024.1343887

**Published:** 2024-04-03

**Authors:** Stine A. Holmboe, Astrid L. Beck, Anna-Maria Andersson, Katharina M. Main, Niels Jørgensen, Niels E. Skakkebæk, Lærke Priskorn

**Affiliations:** ^1^ Department of Growth and Reproduction, Copenhagen University Hospital - Rigshospitalet, Copenhagen, Denmark; ^2^ International Center for Research and Research Training in Endocrine Disruption of Male Reproduction and Child Health (EDMaRC), Copenhagen University Hospital - Rigshospitalet, Copenhagen, Denmark; ^3^ Department of Clinical Medicine, Faculty of Health and Medical Sciences, University of Copenhagen, Copenhagen, Denmark

**Keywords:** cryptorchidism, testicular dysgenesis, prenatal exposure, endocrine disrupting compounds, epidemiology

## Abstract

Congenital cryptorchidism, also known as undescended testis, is the condition where one or both testes are not in place in the scrotum at birth and is one of the most common birth defects in boys. Temporal trends and geographic variation in the prevalence of cryptorchidism from 1% to 9% have been reported in prospective cohort studies. The testes develop in the abdominal cavity and descend to the scrotum in two phases, which should be completed by gestational week 35. Thus, the risk of cryptorchidism is higher in preterm boys. In many cases a spontaneous descent occurs during the first months of life during the surge of gonadotropins and testosterone. If not, the testis is usually brought down to the scrotum, typically by surgery, to increase future fertility chances and facilitate cancer surveillance. The increasing frequency of impaired semen quality and testicular cancer, with which cryptorchidism is associated, represents a concern for male reproductive health in general and a need to understand its risk factors. The risk of cryptorchidism is closely related to gestational factors (preterm birth, low birth weight and intrauterine growth restriction), and especially maternal smoking seems to be a risk factor. Evidence is accumulating that the increasing prevalence of cryptorchidism is also related to prenatal exposure to environmental chemicals, including endocrine disrupting compounds. This association has been corroborated in rodents and supported by ecological studies. Conducting human studies to assess the effect of endocrine disrupting chemicals and their interactions is, however, challenged by the widespread concomitant exposure of all humans to a wide range of chemicals, the combined effect of which and their interactions are highly complex.

## Introduction

Congenital cryptorchidism, also known as undescended testis, is the condition where one or both testes are not in place in the scrotum at birth. This is one of the most common birth defects in newborn boys with a prevalence ranging from 1% to 9% in prospective cohort studies (1, 2), of which around 10% of cases are bilateral (3). Acquired cryptorchidism or ascending testes, where the testis ascends to a higher position after having been in the scrotum and retractile testes, when scrotal testes frequently retract to the groin, are not the focus of this review.

The testes develop in the abdominal cavity. Their descent to the scrotum is often described in two stages with different morphological and hormonal characteristics. The first, transabdominal stage, covering the descend to the internal inguinal ring between gestational week eight and fifteen, is highly dependent on insulin-like peptide 3 (INSL3) from the developing Leydig cells, which acts on the gubernaculum. The gubernaculum attaches the testis to the inner opening of the inguinal canal and creates a pathway for the testicular descent. The second, inguinoscrotal stage, where the testes move to their final position in the scrotum, begins around gestational week 25 and is in most cases completed by the 35^th^ week of gestation. In this stage androgens play a key role (4). The testicular position is usually assessed at birth. In cases of cryptorchidism, the testis may be anywhere along the normal route of descent, in rare cases non-existent (vanishing testes) or ectopic. However, anomalies are most often related to the more complex second stage of testicular descent, while intra-abdominal testes, related to the first stage, are only seen in 5-10% of cases (4).

Around 80% of cases of cryptorchidism spontaneously descend within the first three months after birth to a low scrotal position during the postnatal surge of gonadotropins and testosterone. If this does not happen within the first six months, cryptorchid testes are usually brought down to the scrotum surgically (orchiopexy) (5, 6). This is done to prevent further deterioration of spermatogenic capacity and facilitate testicular cancer surveillance, as cryptorchidism is a risk factor for infertility and testicular cancer (7, 8). Approximately 10% of infertile men have a history of cryptorchidism (9), and based on men from the general population, unselected regarding fertility potential, a history of cryptorchidism was associated with reduced spermatogenic capacity, including reduced testis size, sperm count and inhibin B/FSH ratio (10). A recent meta-analysis estimated a fourfold increased risk of testicular cancer in boys with congenital cryptorchidism treated surgically compared to boys with no history of congenital cryptorchidism (11). However, while orchiopexy is associated with improved fertility chances, it remains unclear whether the risk for testicular cancer is also reduced. Notably, unilateral cryptorchidism should likely be considered a bilateral developmental disease as histopathological abnormalities and a higher testicular cancer risk is also observed in the contralateral non-cryptorchid testis (11).

The increasing frequency of impaired semen quality (12) and testicular cancer (13), with which cryptorchidism is associated, represents a concern for male reproductive health in general and a need to understand its risk factors (14). Here, we give a brief overview of the epidemiology of cryptorchidism, including national and temporal differences in its prevalence and risk factors, including environmental effects.

## Prevalence of cryptorchidism

**Figure 1:**
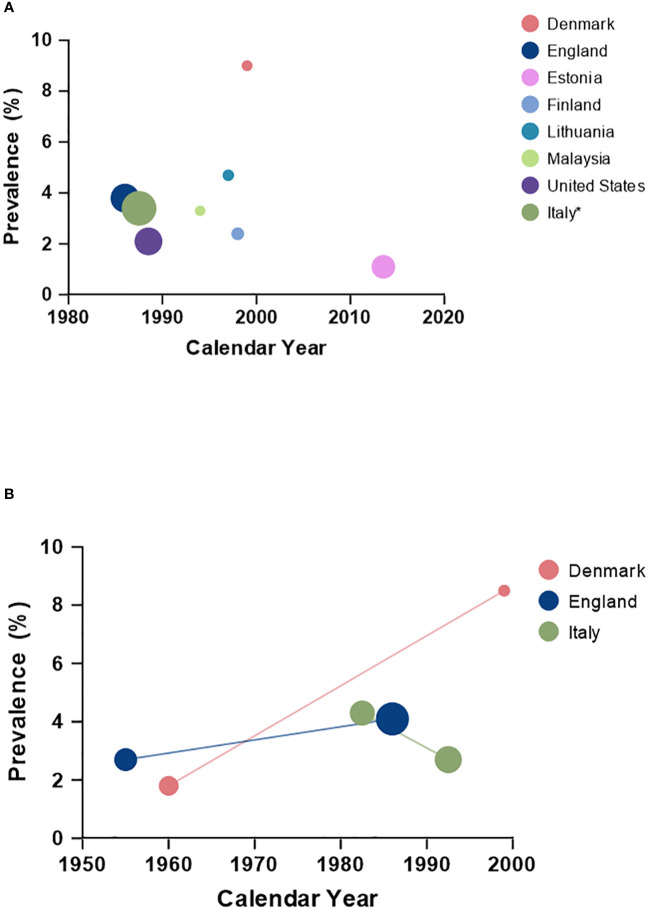
Prevalence (%) of cryptorchidism according to calendar year across countries; **(A)** including boys born term, and **(B)** including countries with comparative data using similar criteria for cryptorchidism within the given country. *The reported number reflects the period from 1978 to 1997.

The reported prevalence of cryptorchidism ranges from 1% to 9% at birth in term, otherwise healthy, boys (1, 2) indicating some variation in this condition which may to some degree be explained by geographical differences. A comparative study of Danish and Finnish newborns using standardized examination protocols showed a four-fold higher risk of cryptorchidism in the Danish newborns compared to the Finnish (9.0% vs. 2.4%, respectively) after adjustment for confounders such as gestational age and birth weight (1). In general, few other European studies have reported the prevalence of cryptorchidism in full-term boys ranging from only 1.1% in Estonia (2), 3.4% in Italy (15), 3.8% in England (16) and 4.7% in Lithuania (17). Non-European studies have reported a prevalence of 2.1% in the United States (18) and 3.3% in Malaysia (19) in full-term boys (**Figure 1A**).

Besides geographical differences, temporal changes in the prevalence of cryptorchidism have been suggested although the registration of cryptorchidism historically has been less reliable which has challenged comparisons over time (20). When applying the exact same clinical definition as previously an increase in the occurrence of cryptorchidism was seen in England from 2.7% in the 1950s to 4.1% in the 1980s in boys with a birthweight of at least 2500g (16, 21). Furthermore, an increase in incidence has been seen in Denmark among boys with normal birth weight when comparing with historical data (1.8% in 1959-61 vs. 8.5% in 1997-01) (1). Finally, data from Italy covering births from 1978-1997 suggest a modest decline in prevalence when stratifying across the time periods: 4.3% in 1978-1987 vs. 2.7% in 1988-1997 (15) (**Figure 1B**). In France the incidence rate of orchiopexy increased from 2.2 per 1000 in 2002 to 2.8 per 1000 in 2014 corresponding to a 36% increase within this period (22). Reasons for these temporal differences remain unclear, however, environmental factors, including differences in prenatal exposures to endocrine disruptors has been suggested as one contributing factor.

Also, ethnic differences in the prevalence of cryptorchidism have been described. A comparative study observed a slightly higher prevalence of cryptorchidism among white Americans compared to black Americans (1.90% vs. 1.55%, p=0.04) (23). In line with this, a comprehensive study from New Zealand observed significant differences in the incidence of cryptorchidism with Maoris having the highest risk followed by Europeans/others, and with Pacific and Asian ethnic groups having the lowest risk (24). Interestingly, the observed differences across ethnic groups mirrored that of testicular cancer in the same populations although the differences were less pronounced. This further supports a common testicular dysgenesis syndrome, i.e. cryptorchidism and testicular cancer are biologically related and both develop prenatally (25, 26).

## Risk factors for cryptorchidism

### Gestational risk factors

Since the last phase of testicular descent occurs in late pregnancy, the increased prevalence of cryptorchidism in preterm boys is to be anticipated and many such cases experience spontaneous descent after birth. Thus, the timing of assessment is an essential factor when comparing data across populations and time. In a recent prospective study of Estonian newborns, a total of 2.1% had cryptorchidism at birth, but the prevalence was 1.1% in full-term newborns, while it was 11.9% among preterm boys. Also low birthweight and intrauterine growth restriction are considered important factors with the prevalence of cryptorchidism being 16.7% among boys of low birthweight and 14% among boys born small for gestational age in the aforementioned study (2, 27). It is challenging to determine whether low birth weight and low weight for gestational age genuinely represent independent risk factors for cryptorchidism – or rather should be considered as proxy indicators linked with the risk of cryptorchidism due to shared underlying risk factors. Several risk factors outlined below also emerge as suggested risk factors for suboptimal *in utero* growth (28, 29). Overall, results from studies on the influence of maternal age on the risk of cryptorchidism are mixed (30). However, maternal age is often associated with other factors that could affect the risk of cryptorchidism, and the reported associations could thus be confounded.

### Maternal health behavior

#### Maternal smoking behavior

Although several smaller studies have reported no association, epidemiological data from various populations have consistently demonstrated an association between maternal smoking and an increased risk of cryptorchidism. This is also the conclusion in four large meta-analyses reporting an overall pooled odds ratio for cryptorchidism between 1.13 and 1.18 in boys of mothers smoking during pregnancy compared to boys of non-smoking mothers (31–34). The magnitude of this risk appears to be dose-dependent, with a higher number of cigarettes smoked per day being associated with a higher risk of cryptorchidism in offspring (35, 36). Furthermore, Jensen et al. found that being preterm modified the association, and a higher risk of cryptorchidism was particularly observed in preterm boys of mothers smoking during pregnancy (37).

#### Maternal alcohol intake

As for maternal smoking, a number of population-based studies have investigated the association between maternal alcohol intake and the risk of cryptorchidism although with inconsistent findings (30). Findings from a meta-analysis including 15 studies and 5,601 cases of cryptorchidism showed no association between maternal alcohol drinking and the risk of cryptorchidism in sons (OR = 0.97, 95% CI: 0.87-1.07, p=0.08) (34). However, when pooling the estimates according to whether the alcohol intake was low (<1 drink/week), moderate (1-5 drinks/week) or high (≥ 5 drinks/week), high alcohol intake was associated with increased risk of cryptorchidism in sons, although insignificantly (OR = 2.74, 95% CI: 0.77-9.80, p=0.12), whereas moderate alcohol intake was associated with an apparent decreased risk of cryptorchidism (OR = 0.89, 95% CI: 0.82-0.96, p<0.01), indicating a non-linear association. The observation of no association between average maternal alcohol intake and risk of cryptorchidism in sons was confirmed in a recently conducted Danish study including more than 80,000 boys with prospectively collected information on maternal alcohol intake during pregnancy (38). However, one or two binge-drinking episodes during pregnancy were associated with a slightly lower risk of cryptorchidism in sons compared with no binge-drinking.

#### Maternal BMI

A Danish study observed a tendency of higher maternal pre-pregnancy BMI being significantly associated with higher cryptorchidism risk in Danish singleton boys (p-trend<0.01) (38). However, there is no consistent evidence suggesting that maternal pre-pregnancy BMI is associated with higher risk of cryptorchidism in sons, and a meta-analysis observed no difference in risk of cryptorchidism for women with BMI > 25 kg/m^2^ compared to women with BMI below this threshold (OR = 1.02, 95% CI: 0.95-1.09), p=0.67) (34). However, this meta-analysis showed that diabetes during pregnancy, for which higher BMI is a risk factor, was associated with increased risk of cryptorchidism (OR = 1.21, 95% CI: 1.00-1.46) (34).

### *In utero* exposure to endocrine disrupting chemicals

The rising prevalence of male reproductive disorders, including cryptorchidism, appearing over a limited time frame, suggests that modern lifestyle plays a role. Specifically, *in utero* exposure to endocrine disrupting chemicals (EDCs) is a suspected contributor. These compounds can disrupt the hormone homeostasis in several ways, including acting on the normal production of hormones, mimicking or blocking their effects, affecting their transport or metabolism, or altering receptor expression. As described, testicular descent is a hormonally dependent event and thus sensitive to disruptions ensued by particularly estrogenic and anti-androgenic effects (39). Establishing associations between EDCs and cryptorchidism in humans is, however, challenging (40), whereas animal studies have clearly shown that *in utero* exposure to environmental chemicals can cause cryptorchidism (41–43). Carefully conducted rodent studies with controlled dose and timing of EDC exposure have demonstrated that anti-androgenic EDC exposure particularly during early fetal development, known as the masculinization programming window, can compromise sufficient androgen action and result in cryptorchidism and other signs of impaired male reproductive function (41, 44). *Ex vivo* studies of human fetal testis tissue manipulating androgen production during different time points of development, support the evidence from animal studies, indicating a critical window of androgen sensitivity observed during gestational week 7-14 in humans (45). During this period, experimentally induced reductions in androgen activity were shown to affect the function and density of several cell types in the human fetal testis with potential implications for the risk of cryptorchidism and future testicular function. Thus, the exact timing of prenatal exposure appears to determine the extent of adverse effects, both in the short term and long term, as the programming of later development and function of the male reproductive organs can be negatively affected (46).

In humans, ecological studies are used to indirectly relate exposures and outcome according to geographic area. Recently, a nationwide French study examined spatial clusters of cases of operated cryptorchidism and found that the incidence differed, with neighboring spatial units tending to show the same risks. Overall, the identified clusters with higher incidences of cryptorchidism were characterized by more dense industrial activities. Such differences could be related to socioeconomic status of the inhabitants but adjusting the spatial analyses for deprivation index did not change the results, suggesting a role of pollution related to former and prior industry (22). Similarly, incidences of cryptorchidism in Korea were shown to be higher in industrialized areas (47). A difference in the chemical burden has also been suggested to contribute to the prior difference in incidence of cryptorchidism observed between Denmark and Finland (48).

Besides ecological studies, an extensive number of epidemiological studies has been conducted assessing the effects of EDC exposure, and the conclusions from such studies are mixed when it comes to specific compounds. These studies aim to determine the level of fetal exposure to EDCs by measuring EDC concentrations in various biological matrices, such as maternal blood, cord blood, maternal urine, amniotic fluid, breast milk, or placenta, or indirectly through self-report of e.g., job exposures. However, such measures often represent an imprecise snapshot of fetal exposure. Furthermore, a major challenge in these studies is the rarity of non-exposed individuals and the fact that humans are continuously exposed to a wide range of chemicals, making it difficult to attribute effects to a single chemical. Most importantly, human exposure studies struggle to account for the common properties and likely interactions between these chemicals, the so-called mixture effects, that animal studies have clearly demonstrated (49). With these limitations in mind, in the following sections we summarize the literature on the epidemiological evidence for associations between the most studied EDCs and the risk of cryptorchidism.

#### Phthalates

Phthalates are used as plasticizers for their durability, transparency, and flexibility. Their appearance in plastics make phthalates ubiquitously present as they exist in a wide range of everyday products such as, but not restricted to, food packaging, pharmaceutical pills, adhesives, cosmetics, grouting, and children’s toys. Their means of entry into the human body are multiple: ingestion, inhalation, intravenous administration (via phthalates in medical devices), and skin absorption. Once taken up, phthalates are rapidly metabolized and are mostly excreted through urine but can also be found in other bodily fluids such as sweat and breast milk. A French study, in which phthalate exposure was indirectly determined through self-reporting of maternal occupational exposure found a tendency of higher prevalence of cryptorchidism among mothers with self-reported phthalate exposure compared to mothers with no self-reported phthalate exposure although the numbers were low (50). In addition, a study assessing maternal urine concentrations of phthalates collected during pregnancy (mean 28.6 gestational week) observed a significant association between higher concentrations of mono-2-ethylhexyl phthalate (MEHP) and cryptorchidism (51). However, the latest systematic review conducted in 2021 included nine studies assessing the impact of phthalates on male reproductive health, where all studies but one (51) found no significant association between phthalate exposure levels and cryptorchidism (52). This is moreover supported by a recent study published in 2022 utilizing maternal urine spot samples from each trimester of 1059 Dutch mothers, in which no association was found between prenatal phthalate exposure and cryptorchidism (53).

#### Bisphenols

Bisphenols are utilized in polycarbonate plastics, epoxy resins and thermal paper (54). Bisphenols are widely found in food packaging, but can also be detected in building materials, toys, cash register receipts, vinyl flooring and cosmetic products. Humans are orally and topically exposed to bisphenols, but also indirectly through the environment in the form of indoor dust (55) and soil pollution (56). Bisphenols have been measured in plasma, urine, cord blood and follicular fluid (57–59). A case-control study utilizing maternal serum concentrations of bisphenol A (BPA) of 330 mothers observed that the chemical was associated with a significant increase of cryptorchidism in sons. The same study furthermore observed that the significant association persisted when restricting their analysis to infants born full-term and not of low birth weight (60). A cohort study utilizing placentas to investigate the risk of urogenital malformations (defined as cryptorchidism and hypospadias) in 668 mother-son pairs observed that placentas within the highest tertile of BPA concentrations were associated with a higher risk of cryptorchidism/hypospadias (61). A Polish study observed that boys with cryptorchidism had significantly higher levels of conjugated BPA and total BPA in serum, however, notably, BPA levels were measured in samples taken prior to surgery rather than prenatally, which challenges any causal inferences as bisphenols are not considered to be persistent (62).

#### Parabens

Parabens are antifungal and antibacterial agents and are therefore widely applied in cosmetics, pharmaceuticals and in processed food. Due to their application in toiletry products, humans are commonly exposed to parabens through dermal application. Parabens permeate through the skin and have been detected in urine, breast milk, blood, and adipose tissues (63–65). In a cohort study utilizing placentas to ascertain paraben exposure and risk of urogenital malformations (defined as cryptorchidism and hypospadias) it was observed that placentas within the highest tertile of propyl-paraben were associated with higher odds of cryptorchidism/hypospadias. This association moreover persisted when including propyl-paraben as a continuous variable (61). In contrast, a study of 334 male infants found no association between cryptorchidism and propyl-paraben measured in maternal serum (60).

#### Pesticides

Pesticides are chemicals utilized to control pests to protect crop, preserve foods and eliminate illnesses spread by vectors. They have various functions such as herbicides, insecticides, avicides and fungicides, and are massively applied in agriculture and gardening. Humans are exposed to pesticides either directly, by using them on plants, or indirectly through consumption of crops and ground water. Pesticides are present in human serum (66), urine (67), adipose tissue (68) and amniotic fluid (69). In a Danish study assessing the self-reported prenatal pesticide exposure through occupation in four cohorts consisting of 1468 mother-son pairs, women occupationally exposed to pesticides had a higher risk of having sons with cryptorchidism than non-exposed women. Furthermore, an increased risk was observed for two out of the four cohorts compared to the background population, but this association was not seen in the combined cohort analysis (70). In a separate case-control study utilizing breast milk samples of Danish and Finnish mothers, 17 of 21 measured organochlorine pesticides were detected at slightly higher median concentrations in breast milk of mothers with cryptorchid sons than those with healthy sons. In addition, the combination of the eight most abundant pesticides were significantly higher in mothers of boys with cryptorchidism than those with healthy boys (71). In a study of 1326 Norwegian mother-son pairs in which exposure to organochlorine pesticides was ascertained in breast milk, only β-Hexachlorocyclohexane (β-HCH) was associated with cryptorchidism (72). A Spanish study that ascertained pesticide exposure in placenta tissues amongst cases of cryptorchidism and hypospadias together observed a higher detection rate of various pesticides in cases (72%) versus controls (54%), as well as an increased risk of the urogenital malformations if the mother’s occupation was in agriculture compared to other occupations. The study moreover observed increased risk of urogenital malformations associated with higher levels of some of the measured pesticides (73). In contrast, some studies have observed no associations between prenatal exposure to pesticides and cryptorchidism (74–77).

#### Poly- and perfluoroalkyl substances

Poly- and perfluoroalkyl substances (PFAS) are a group of chemicals used to make products resist heat, stains, oil, water, and grease. Due to their practicality, PFAS have been used in a wide range of everyday products, such as building materials, textiles, impregnation sprays, cookware, and fire-fighting foams. Their use in some products have since been restricted, yet due to their resistance to biodegradation through their carbon-fluorine bond, they can still be found accumulating in the environment (78, 79) and in the human body (80). Humans are mainly exposed to PFAS through drinking water, inhalation, and food (81). In a nested case-control study in 215 Danish and Finnish boys, perfluorooctanoic acid (PFOA) and perfluorooctanesulfonic acid (PFOS) was detected in all cord blood samples, however, no association was seen in relation to cryptorchidism (74). Similar findings of no association were also observed in a Danish study of 270 cryptorchidism cases and amniotic fluid levels of PFOS (82).

#### Polychlorinated biphenyls

Polychlorinated biphenyls (PCBs) have been widely applied in industrial applications due to their low flammability making them attractive to use as additives in the production of e.g., coolants, building materials and flame retardants. Humans are exposed to PCBs through inhalation and diet (83) with PCBs being detectable in serum and cord blood and showing accumulation in the brain (84–86). A Danish/Finnish case-control study of 130 breast milk samples observed that nine PCBs were higher in cryptorchid Danish cases than controls, whereas this was not seen in the Finnish cohort (48). A study utilizing postal addresses to identify PCB-contaminated apartments as a proxy of exposure reported that pregnant women had a 73% higher risk of giving birth to a son with cryptorchidism compared to unexposed women (87). Furthermore, a French study of 164 mother-son pairs utilizing colostrum samples observed that cases had higher individual scores of PCB than controls. Conversely, other studies have found no association between *in utero* exposure to PCBs and cryptorchidism (88–90).

## Conclusion

To summarize, the incidence of cryptorchidism shows both regional and temporal differences. The risk in cryptorchidism is closely related to gestational factors (preterm birth, low birth weight and intrauterine growth restriction), and especially maternal smoking seems to be a risk factor. The increasing prevalence of cryptorchidism is likely also related to prenatal exposure to environmental chemicals, including endocrine disrupting compounds. This association has been corroborated in rodents and supported by ecological studies. Conducting human studies to assess the effect of endocrine disrupting chemicals and their interactions is challenged by the widespread concomitant exposure of all humans to a wide range of chemicals, the combined effect of which and their interactions are highly complex.

## Author contributions

SH: Writing – original draft. AB: Writing – original draft. A-MA: Writing – review & editing. KM: Writing – review & editing. NJ: Writing – review & editing. NS: Writing – review & editing. LP: Writing – original draft.
